# Relationship between nocturnal serotonin surge and melatonin onset in rodent pineal gland

**DOI:** 10.1186/1740-3391-4-12

**Published:** 2006-09-27

**Authors:** Tiecheng Liu, Jimo Borjigin

**Affiliations:** 1 Department of Molecular and Integrative Physiology, University of Michigan Medical School, Ann Arbor, MI 48109, USA

## Abstract

**Background:**

We have recently reported dynamic circadian rhythms of serotonin (5-HT, 5-hydroxytryptamine) output in the pineal gland of rat, which precedes the onset of N-acetylserotonin (NAS) and melatonin secretion at night. The present study was aimed at investigating in detail the relationship between 5-HT onset (5HT-on) and melatonin onset (MT-on) in multiple strains of rats and comparing them with those of hamsters.

**Methods:**

Animals were maintained in chambers equipped with light (250 lux at cage levels) and ventilation in a temperature-controlled room. Following surgical implantation of a microdialysis probe in the pineal gland, animals were individually housed for on-line pineal microdialysis and for automated HPLC analysis of 5-HT and melatonin. Animals were under a light-dark cycle of 12:12 h for the duration of the experiments.

**Results:**

All animals displayed dynamic 5-HT and melatonin rhythms at night. In all cases, 5HT-on (taken at 80% of the daily maximum levels) preceded MT-on (taken at 20% of the daily maximum levels). Within the same animals, 5HT-on as well as MT-on across multiple circadian cycles exhibited minimum variations under entrained conditions. Large inter-individual variations of both 5HT-on and MT-on were found in outbred rats and hamsters under entrained conditions. In comparison, inbred rats displayed very small individual variations of 5HT-on and MT-on. Importantly, we have uncovered a species-specific relationship of 5HT-on and MT-on. 5HT-on of rats, regardless of the strain, preceded MT-on of the same rats by 50 min. In contrast, 5HT-on of hamsters led MT-on by as much as 240 min. Thus, while a constant relationship of 5HT-on and MT-on exists for animals of the same species, the relative timings of 5HT-on and MT-on differ between animals of different species.

**Conclusion:**

These results suggest that both 5-HT and melatonin could serve as reliable markers of the circadian clock because of their day-to-day precision of onset timings within the same animals or within individuals of the same strain or same species. The results also demonstrate that data for MT-on cannot be compared directly between different species, and that 5HT-on may be a more reliable circadian marker when data from animals of different species are compared.

## Background

Circadian rhythm studies have utilized the onset phases of rhythmic outputs, such as locomotor activities of laboratory rodents [1], temperature, and melatonin secretion [2,3], as markers of the circadian clock. The reliability and consistency of these circadian output markers are essential for an accurate pacemaker analysis across multiple circadian cycles of the same individuals, between different individuals of the same animal strain, between different strains of the same animal species, and between individuals of different species [4]. This study analyzes circadian characteristics of pineal 5-HT and melatonin secretion in rats and hamsters under entrained conditions.

The pineal gland of all vertebrate animals produces melatonin from 5-HT in response to the norepinephrine released from the superior cervical ganglion (SCG) [5,6]. The production and secretion of 5-HT and melatonin are regulated by the circadian clock situated in the suprachiasmatic nuclei, and are elevated at night in rats [7]. Because of the tight association of pineal melatonin release with the clock activity, melatonin has been regarded as an accurate marker of the circadian pacemaker for both animal and human circadian rhythm studies [8-12]. In human studies, for example, the onset timing of melatonin release at night has been used extensively to determine the phase angle of circadian pacemaker entrainment [13]. Comparative analysis of MT-on from different individuals is conducted routinely in human studies, which is linked to circadian traits such as morningness and eveningness [13]. Such analyses are largely missing from animal studies for inter-cycle, inter-individual, inter-strain, and inter-species differences of MT-on. This is due to difficulties with high-resolution sampling and analysis of melatonin in small laboratory rodents using conventional methods. These difficulties are now overcome with our pineal microdialysis technique, which allows high-resolution analysis of pineal melatonin secretion in individual animals for extended periods in real time [2,3,7,14]. Using this technique, this study is designed to provide detailed profiles of 5-HT and melatonin release under entrained conditions in rodents.

Pineal melatonin is produced at night from 5-HT, which is synthesized within the pineal gland and displays dynamic circadian rhythms of both synthesis and release [7]. Unlike melatonin, whose synthesis in rodents requires transcriptional activation of the key enzyme serotonin N-acetyltransferase [15], the nocturnal surge of 5-HT production and release is controlled posttranscriptionally in the pineal gland [7]. Earlier data indicate that both synthesis and release of 5-HT are triggered by norepinephrine released from the SCG nerve terminals [7]. Compared to the lag time of about 1 h for melatonin output, there appears to be a minimum delay for 5-HT release when norepinephrine is directly infused into the pineal gland via microdialysis tubing [7,14]. These data indicate that the surge of 5-HT release in the night pineal gland broadcasts the timing of norepinephrine release from the SCG and, hence, the arrival timing of central circadian clock signals. Initiation of melatonin production, however, could depend on pineal mechanisms downstream of the 5-HT surge, which could differ between individual animals of the same strain, be unique to each strain of rats, or be specific to different species of animals. In this paper, secretion profiles of 5-HT and their relationship with melatonin are studied across multiple circadian cycles in multiple animals of the same strains, in multiple outbred as well as inbred strains of rats, and in both rats and hamsters.

## Methods

### Animals

Adult (2–4 months) male animals of several strains of outbred and inbred rats, and outbred hamsters were analyzed in this study. Sprague Dawley (SD, outbred) rats, Wistar rats that are transgenic for per1-luc constructs (Wistar, outbred), PVG rats (inbred), Lewis (LEW, inbred) rats, and Syrian hamsters (outbred) were used. The transgenic Wistar rats are kindly provided by Dr. Michael Menaker. All rats, except the Wistar rats, were purchased from Harlan (Indianapolis, IN), whereas the Syrian hamsters were from Charles River Laboratory (Wilmington, MA). Animals were housed in a temperature-controlled chamber with a light and dark (LD) cycle of 12:12 h (lights on at 6 am).

Prior to on-line analysis of 5-HT and melatonin, animals were implanted with microdialysis probes directly in their pineal glands as described [14], and allowed to recover for 1–3 days. Following recovery, animals were transferred to the dialysis chamber into their individual cages. The dialysis chamber was temperature-controlled, ventilated, and light proof. Illumination was supplied by white fluorescent lamps (250 lux at cage level).

### Surgery

Detailed protocols of the surgical implantation of pineal microdialysis probes, on-line microdialysis, and automated HPLC analysis were as described [14]. Briefly, following deep anesthesia, the animal's head was positioned in a stereotaxic instrument. A circular skull opening was created using a dental disk drill, and the pineal gland was exposed by slightly lifting the confluence of the superior sagital sinus along with the dura matter with a hook fixed to the stereotaxic frame. The tip of the guide cannula (CMA/MD, N. Chelmsford, MA, USA) was then positioned adjacent to either side of the exposed pineal before the skull was closed with dental cement. The operated animals were allowed to recover 1–3 days before experimentation.

### Microdialysis

Pineal microdialysis was carried out as follows. Immediately before sampling, animals were anesthetized with halothane briefly; the stylet (or dummy probe) was replaced with a microdialysis probe (CMA12, 20 kD cut-off, membrane length 4 mm) (CMA/MD, N. Chelmsford, MA) and fixed with plastic glue. The dialysis probe was continuously infused via microbore PEEK tubing (0.65 mm OD, 0.12 mm ID) at a flow rate of 2 μl/min with artificial cerebral spinal fluid (CSF; Harvard Holliston, MA). Samples were collected at 10 min intervals via PEEK tube into a 20-μl loop of a Pollen 8 automatic injector (BAS, West Lafayette, IN), which was on-line with the high-performance liquid chromatography (HPLC) system. The sample loop was set to be retained in the load position during the 10-min cycles and was automatically switched to the injection position briefly, after which the cycle was repeated. Animals were linked to the apparatus for dialysis through a quartz dual channel swivel (Instech, Boston, MA) to prevent the tubing from entanglement.

### HPLC analysis

The analytical condition for the simultaneous detection of 5-HT and melatonin was based on Drijfhout et al. [16] with minor modifications. A Shimadzu pump (Shimadzu, Columbia, USA) was used in conjunction with a Shimadzu fluorescence detector (FD, excitation: 280 nm, emission: 345 nm). Samples were injected into the system through a Valco injection valve with a BAS controller and subsequently separated on a reversed phase C18 column (250 × 4.6 mm; Supelco Bellefonte, PA), set at a constant temperature of 30°C using a Shimadzu column heater, controlled by a Shimadzu system controller. The mobile phase consisted of a mixture of 10 mM sodium acetate, adjusted to a pH of 4.5 with concentrated acetic acid, 0.01 mM Na_2_-EDTA, 500 mg/l heptane sulfonic acid and 22% (v/v) acetonitrile. The flow rate of the HPLC pump was set at 1.5 ml/min throughout the experiment. Standard solutions of melatonin were used to calibrate the system. The automated control of the HPLC system, the programming of the flow rate, and the handling and storage of the chromatograms were carried out with an external computer using the Shimadzu Class-VP 5.03 chromatography software.

## Results

**Figure 1:**
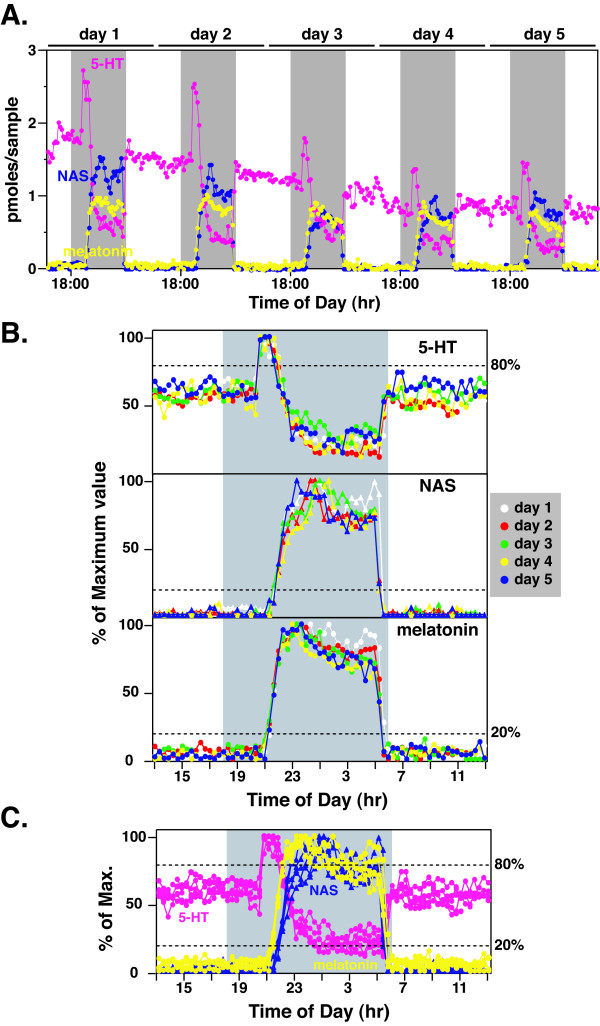
**Circadian rhythms of pineal 5-HT, N-acetylserotonin (NAS), and melatonin in a LEW rat over a 5-day period**. **A**. 5-HT (pink), NAS (blue), and melatonin (yellow) profiles of a single LEW rat are shown for 5 consecutive cycles. In this and all subsequent figures, the shaded gray areas represent the dark period (18:00 h to 6:00 h). The daily boundaries were arbitrarily set at 13:00 h for ease of comparison. **B**. 5-HT (top panel), NAS (middle panel), and melatonin (bottom panel) data from each 24 h period (13:00 h to 13:00 h) were normalized to its nocturnal maximum values and superimposed together. Pineal secretory activities of 5-HT, NAS, and melatonin in different cycles were represented with different colors, and the values of 5-HT, NAS, and melatonin from the same cycle were represented with the same colors. The horizontal dashed lines represent onset phase of 5-HT (top panel, 80%), NAS (middle panel, 20%), and melatonin (bottom panel, 20%). **C**. Profiles of 5-HT (pink), NAS (blue), and melatonin (yellow) of the rat shown in B were superimposed in the same panel for a clearer comparison.

Secretion profiles of 5-HT, NAS, and melatonin were studied in a LEW rat for multiple circadian cycles (Figure 1), using the long-term pineal microdialysis technique [14]. During each cycle, 5-HT secretion surged at early night, which precedes the onset of both NAS and melatonin (Figure 1A). The peak secretion of 5-HT displayed a gradual decrease over the first 3-day period, and remained stable for the rest of the sampling period (3 wks, data not shown). Despite the difference in the amplitude of 5-HT, NAS, and melatonin secretion, however, the timing of all three circadian products was remarkably consistent from day-to-day, when normalized to their nocturnal maximum levels. No detectable differences were seen for 5HT-on (assessed at 80% of the maximum levels), NAS onset (20%), and MT-on (20%) over the experimental period (Figure 1B). The same consistent pattern was obtained for NAS and melatonin offset (decline phase), except for the first day of analysis (day 1). Similar data were obtained from all animals analyzed (data not shown). When all three products were normalized to its nocturnal maximum levels and compared side-by-side, a 50 min of lag between 5HT-on and MT-on was consistently found during the entire sampling period (Figure 1C). Interestingly, NAS output was delayed for about 20 min compared to the MT-on.

**Figure 2:**
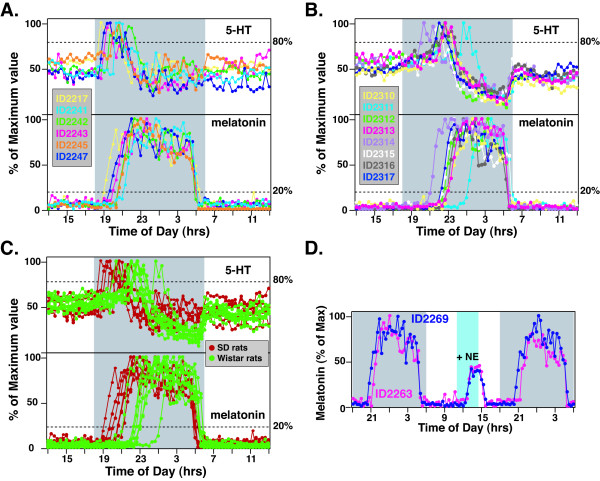
**Circadian profiles of pineal 5-HT and melatonin secretion from multiple *outbred *SD and Wistar rats**. **A**. Entrained profiles of 5-HT (upper panel) and melatonin (lower panel) secretion from 6 SD rats. All data points were normalized to the nocturnal peak values of each circadian cycle. Each rat is represented by a different color. 5-HT and melatonin secretion from the same rat is shown in the same color. **B**. Secretory activities of 5-HT (upper panel) and melatonin (lower panel) from 8 Wistar rats. **C**. Comparison of 5-HT (upper panel) and melatonin (lower panel) secretion profiles from SD (red) and Wistar (green) rats. **D**. Phase angle difference in MT-on is independent of pineal mechanisms. Norepinephrine (NE, 20 uM) was infused directly into the pineal gland of the rats ID2263 (pink) and ID2269 (blue) for 3.5 hrs (11:00 h-14:30 h; blue shaded area). Data for melatonin secretion were normalized to the nocturnal maximum of the first night.

In contrast to the consistent day-to-day profiles of pineal circadian secretory products within the same animal (Figure 1), large inter-individual differences were found when multiple rats from the same outbred strains were analyzed and compared (Figure 2). In this and all subsequent figures, each circadian profile of either 5-HT or melatonin was the representative pattern of each individual animal under LD (12:12 h) entrained conditions. Six individual SD rats were analyzed for entrained profiles of 5-HT and melatonin secretion (Figure 2). The onset timing of 5-HT ranged from 25 to 131 min, while that of melatonin varied from 60 to 188 min (Figure 2A). Outbred Wistar rats, transgenic for per1-luc construct [17], were also analyzed for their profiles of 5-HT and melatonin secretion. Unlike the SD rats, the Wistar rats displayed delayed onset of both 5-HT (120–387 min) and melatonin (185–425 min) secretion. The strain-specific characteristics of SD and Wistar rats were clearly seen when the secretory profiles of 5-HT and melatonin were compared side-by-side (Figure 2C). The time interval between 5HT-on and MT-on, however, appeared to be maintained for each individual animal in both SD and Wistar rats (Figures 2A and 2B; see below).

To rule out the possibility that differences in MT-on are due to differential mechanisms of melatonin activation within the pineal, we stimulated melatonin production using norepinephrine via direct infusion into the pineal through microdialysis tubing during the daytime. Two SD rats with different phase angles (time intervals between the darkness onset and the 5HT-on and MT-on) were chosen for this study, and their melatonin patterns were superimposed in Figure 2D. While the MT-on between rat ID2269 and rat ID2263 differed by 60 min (left and right side of the panel), no difference in activation kinetics of pharmacologically stimulated melatonin production (blue shaded area) was observed between the two rats (Figure 2D). We have also infused norepinephrine into the pineals of 6 additional rats that exhibited variable phase angles of MT-on via microdialysis tubing. All tested rats displayed activation time course for melatonin identical to those shown in Figure 2D (data not shown). These data demonstrate that the phase angle differences in MT-on of SD rats are not caused by differences in responsiveness within the pineal gland, but are rather controlled by a central mechanism, mostly by the circadian pacemaker in the brain.

**Figure 3:**
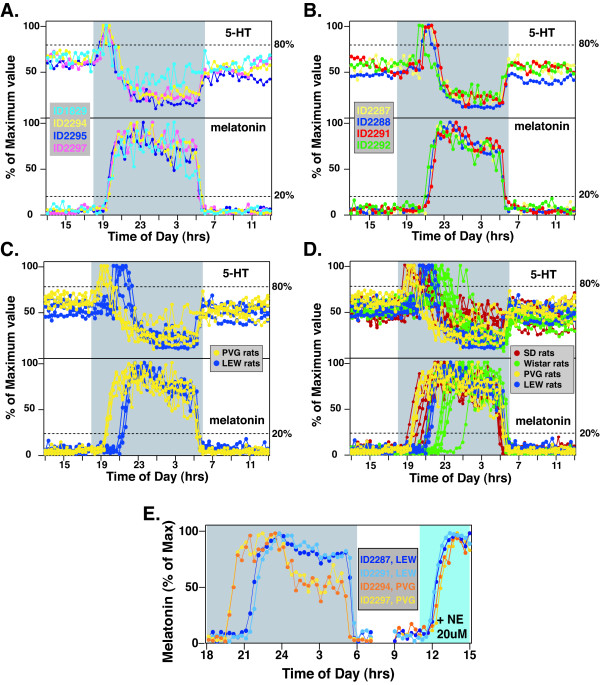
**Circadian profiles of pineal 5-HT and melatonin secretion from multiple *inbred *PVG and LEW rats**. **A**. Entrained profiles of 5-HT (upper panel) and melatonin (lower panel) secretion from 4 PVG rats. All data points were normalized to the nocturnal peak values of each circadian cycle. Each rat is represented by a different color. 5-HT and melatonin secretion from the same rat is shown in the same color. **B**. Secretory activities of 5-HT (upper panel) and melatonin (lower panel) from 4 LEW rats. **C**. Comparison of 5-HT (upper panel) and melatonin (lower panel) secretion profiles from PVG (yellow) and LEW (blue) rats. **D**. Comparison of 5-HT (upper panel) and melatonin (lower panel) secretion profiles from all 4 strains of rats: SD (red), Wistar (green), PVG (yellow), and LEW (blue) rats. **E**. Phase angle difference in MT-on between PVG and LEW rats is independent of pineal mechanisms. Norepinephrine (NE, 20 uM) was infused directly into the pineal gland of 2 PVG (ID2294 and ID2297) and 2 LEW (ID2287 and ID2291) rats for 4 hrs (11:00 h-15:00 h; blue shaded area). Data for melatonin secretion at night were normalized to the nocturnal maximum, whereas data between 9:00 h and 15:00 h were normalized to the maximum levels stimulated by the norepinephrine (13:00 h-14:00 h). Compared to PVG rats, appearance of melatonin following norepinephrine stimulation was slightly delayed (10–20 min). This difference was due to the time needed to change drug-filled syringes.

In addition to the outbred animals, we have analyzed the inbred rats of PVG and LEW strains for their 5-HT and melatonin secretion profiles using on-line pineal microdialysis. Multiple rats (n = 4) of each inbred strain were analyzed following entrainment in LD 12:12 h, and the entrained 5-HT and melatonin profiles of each rat were displayed as a percent of the maximum at night. As shown in Figure 3A and 3B, the phase angles of both 5HT-on and MT-on for either PVG rats or LEW rats are remarkably consistent between individuals of the same strain. For PVG rats (Figure 3A), the 5HT-on ranged from 38 to 52 min (upper panel), while the MT-on clustered between 42 min to 68 min among the 4 rats (lower panel). For LEW rats (Figure 3B), the 5HT-on ranged from 130 to 172 min (upper panel), and the MT-on spanned from 159 to 179 min (lower panel). Thus, within each individual inbred strain, inter-individual variations of 5HT-on and MT-on are relatively small.

When the two inbred strains are compared with each other, however, strain-specific characteristics of 5-HT and melatonin secretion were apparent (Figure 3C). Both 5HT-on and MT-on of PVG rats preceded that of LEW rats for 110 min in LD12:12h. Data from inbred rats were further compared with those from outbred rats, which showed that the inbred rats displayed much smaller inter-individual differences in phase angles of 5HT-on and MT-on (Figure 3D). The strain-specific phase angles of 5HT-on and MT-on for PVG and LEW rats represent the extreme types of 5HT-on and MT-on found in SD rats, and are much earlier than those of the Wistar rats (Figure 3D).

As with SD rats in Figure 2D, we sought to determine if the phase angle differences of MT-on in the inbred PVG and LEW rats are due to a difference in the central clock activity or, alternatively, differential responses within the pineal. If the differences are due to intrinsic differences in the pineal, direct stimulation of the pineal should cause differences in melatonin secretion delays between strains. Norepinephrine (NE, 20 μM) was infused directly into the pineal glands of two PVG rats (ID2293 and ID2297) and two LEW rats (ID2287 and ID2291) during the daytime (Figure 3E). While the MT-on at early night differs by about 2 h between the two strains (left panel; Figure 3E), little difference was found when melatonin was induced directly by norepinephrine infusion into the pineal glands of these rats (right panel; Figure 3E). These data strongly indicate a central origin of the phase angle difference between the two strains.

**Figure 4:**
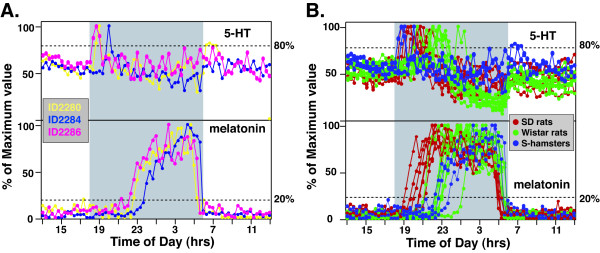
**Circadian profiles of pineal 5-HT and melatonin secretion from multiple *outbred *Syrian hamsters**. **A**. Entrained profiles of 5-HT (upper panel) and melatonin (lower panel) secretion from 3 Syrian hamsters. All data points were normalized to the nocturnal peak values of each circadian cycle. Each hamster is represented by a different color. 5-HT and melatonin secretion from the same hamster is shown in the same color. **B**. Comparison of 5-HT (upper panel) and melatonin (lower panel) secretion profiles from outbred animals. Data from SD rats (red), Wistar rats (green), and hamsters (blue) are superimposed to show the relative timing of 5-HT and melatonin secretion.

5-HT and melatonin secretion profiles were also studied in Syrian hamsters in LD 12:12 h conditions (Figure 4). 5HT-on and MT-on ranged from 17–109 min and 251–344 min, respectively (Figure 4A). We compared the secretion patterns of 5-HT and melatonin of the hamsters with those of rats (Figure 4B). While the hamsters displayed very early 5HT-on (upper panel) compared to both SD and Wistar rats, their MT-on was much delayed. Thus, the interval between 5HT-on and MT-on for hamsters was 4 h, while the lag between 5HT-on and MT-on of rats was about 1 h. A difference of 3 h in MT-on, following the onset of 5-HT surge, exists between the rats and hamsters.

**Figure 5:**
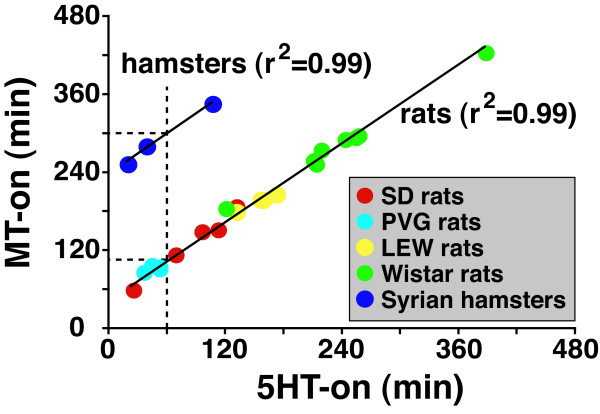
**Comparison of the relative timing of 5-HT onset (5HT-on) and melatonin onset (MT-on)**. SD: red dots (n = 6), PVG: blue dots (n = 4), LEW: yellow dots (n = 4), Wistar: green dots (n = 8), Syrian hamsters: blue dots (n = 3). The 5HT-on represents the intervals (min) between the dark onset (18:00 h in all animals) and the early 5-HT surge at 80% of the nocturnal maximum levels. The MT-on indicates the time interval (min) between the dark onset (18:00 h) and the melatonin onset at 20% of the nocturnal maximum levels. The vertical dashed line represents the 5HT-on at 60 min, whereas the horizontal dashed lines represent values of MT-on from hamsters (upper line, 300 min) and rats (lower line, 110 min) when 5HT-on is 60 min.

The relationship of the onset of 5-HT secretion with the onset of melatonin release was analyzed for each of the animals described above (Figure 5). A linear correlation with r^2 ^of 0.99 was found between 5-HT and melatonin secretion onset for all individuals studied (Figure 5). Thus, regardless the individual differences within the same rat strains, or the inter-strain differences in rats, the MT-on is in a fixed relationship with the 5HT-on within the same species. The relationship of 5-HT surge and melatonin onset, however, appears to be unique for individual animal species: while the lag between 5HT-on and MT-on was always about 1 h (50 min) in rats; the lag in hamsters, however, was 4 h (Figure 5).

## Discussion

Onset timing of circadian behaviors (sleep-wake, activity-rest, food intake etc.) and daily physiological events (pineal 5-HT/NAS/melatonin secretion, cortisol or growth hormone release) represents one of the most important characteristics of the circadian pacemaker. Earlier onset of melatonin secretion and sleep in humans, for instance, is indicative of a shorter circadian clock period in individuals with the Advanced Sleep Phase Syndrome, compared with normal individuals [18]. Because of its tight association with the pacemaker activities, melatonin is considered the most reliable marker of the circadian pacemaker, especially in human circadian studies [8]. Recent development of the long-term pineal microdialysis technique from our laboratory has opened possibilities of using pineal circadian hormones to mark the central pacemaker in animal studies [2,3,7,14]. This paper represents the first detailed analysis of inter-cycle (of the same individual), inter-individual, inter-strain, and inter-species characteristics of pineal 5-HT and melatonin secretion in rodents.

### Inter-cycle rhythms of 5-HT and melatonin secretion

When pineal secretory activity was analyzed in the same rats over multiple circadian cycles, we have found that the onset as well as offset timings of 5-HT, NAS, and melatonin secretion are identical from cycle to cycle with no detectable daily fluctuation. This remarkable degree of consistency suggests that circadian pacemaking may be a far more precise process than previously recognized. The precisely controlled onset and offset of melatonin (and 5-HT) secretion permits detection and in-depth analysis of small changes in phase angles of circadian pacemaker following shifts of the LD cycle, or pharmacological perturbations.

### Inter-individual variations of 5-HT and melatonin secretion

Large inter-individual variations in 5HT-on and MT-on were identified in the outbred SD rats and Syrian hamsters. Similar to an earlier study [19], a large inter-individual variation of MT-on was also found for Wistar rats in this study. Consistent with PVG and LEW being inbred animals, a much smaller inter-individual variation in 5HT-on and MT-on was found in these strains of animals. Thus, the inbred strains are more suitable for circadian studies that require a group of similar animals. In each of these animals, however, the inter-individual variations in MT-on were directly and invariably associated with the variations in 5HT-on, which is controlled by the timing of norepinephrine release at night [7]. These results support the notion that the timing (or phase angle) of MT-on in these animals is controlled by a central circadian clock via regulation of the timing of norepinephrine release. We plan to demonstrate this hypothesis by directly measuring norepinephrine release in our future studies.

### Inter-strain variations of 5-HT and melatonin secretion

In addition to the inter-individual variations described earlier, we have found strain specific characteristics of 5-HT and melatonin secretion. Compared to the SD rats, whose MT-on varied between 60–188 min, the Wistar rats showed MT-on of 185–425 min. Thus, the SD rats possess advanced phase angles of MT-on compared to the Wistar rats. Similarly, PVG rats display earlier onset of 5-HT and melatonin compared to the LEW rats. Importantly, however, the interval between 5HT-on and MT-on remained constant in each individual of the same strain, and in each strain of rats. These results suggest that either 5HT-on or MT-on can serve *interchangeably *as a reliable marker of the circadian clock when different individuals of the same strain or different strains of the same species of rats are compared.

### Inter-species variations of 5-HT and melatonin secretion

In contrast to the 1 h lag time between 5HT-on and MT-on in rats, a 4 h delay of MT-on from the onset of 5-HT release was found in hamsters. The relatively late MT-on of hamsters is possibly due to a delayed activation of serotonin N-acetyltransferase [20], the key enzyme in melatonin synthesis [5,21]. Compared to rats, however, the hamsters displayed relatively early onset of 5-HT surge. Thus, the interval between 5HT-on and MT-on is likely to be unique to individual species.

### Phase angle of 5-HT/melatonin onset and circadian period

The entrained phase angle is thought to be a function of the difference between the environmental period and the circadian period [1,22]. The smaller the period, the earlier the phase angle becomes. In human individuals with a 3.5 h advanced onset of melatonin and sleep [18], it was found that the circadian period was 23.3 h, which is about 1 h shorter than the average period of human individuals [23] with a normal phase of MT-on. Circadian period of the SD rats (24.05–24.13 h) [24] is known to be shorter than that of Wistar rats (24.43 h) [25], when locomotor onset was studied. Our own analysis of the SD rats, using MT-on as a marker, has demonstrated period value of 24.07–24.22 h [3]. Consistent with these earlier data, the MT-on of the SD rats was more advanced than that of the Wistar rats in this paper. Similarly, the 5HT-on was also earlier in SD rats than in Wistar rats. These results suggest that when animals of the same species are compared, either 5HT-on or MT-on can be used reliably as reference marker of the pacemaker. When animals from different species are compared, however, our data suggest that MT-on cannot serve as inter-species reference marker, as mechanisms that govern melatonin synthesis differ between different species [21]. Earlier studies have determined the circadian period of Syrian hamsters to be shorter than that of rats, at or very close to 24 h [1]. To be consistent with the period data of rats, therefore, the entrained phase angle of a circadian marker in hamsters ought to be more advanced than that of rats. The onset phase of 5-HT appears to be uniquely suited for this purpose, as their onset timing was earlier than those from rats in our studies.

Unlike locomotor and temperature rhythms, which display dramatic differences between diurnal and nocturnal animals [26], melatonin is secreted in greater abundance at night in all vertebrate animals. This feature, combined with the fact that melatonin secretion is tightly linked to the central clock activities [27], makes melatonin an attractive additional marker for animal models of circadian rhythm studies [8]. Using the pineal microdialysis technique, we have found that both NAS and 5-HT from the pineal gland, in addition to melatonin, can potentially serve as valuable markers for pacemaker analysis. Findings in this study demonstrate that while melatonin is an excellent marker of the circadian pacemaker when inter-cycle, inter-individual, and inter-strain rhythms of the same animal species are concerned, caution must be used when melatonin data are compared among different species. When the pineal microdialysis technique is utilized in a rhythm study, our data demonstrate that onset of nocturnal surge in 5-HT release may be more suited for inter-species comparative analysis of circadian rhythms. Our future studies will include investigating whether the 5-HT and melatonin onset phases are directly correlated with animal's circadian periods, and if variations of 5-HT/melatonin onset are associated with circadian variations in behavior and physiology in animal model systems.

## Competing interests

The author(s) declare that they have no competing interests.

## Authors' contributions

TL performed all experiments, which include surgeries, sample collection via microdialysis, and HPLC sample analyses. JB directed the study, analyzed data, and wrote the manuscript. Both authors read and approved the final version of the article.

## References

[B1] Pittendrigh CS, Daan S (1976). A functional analysis of circadian pacemaker in nocturnal rodents: I. The stability and lability of circadain frequency.. J Comp Physiol A.

[B2] Liu T, Borjigin J (2005). Reentrainment of the circadian pacemaker through three distinct stages. J Biol Rhythms.

[B3] Liu T, Borjigin J (2005). Free-running rhythms of pineal circadian output. J Biol Rhythms.

[B4] Daan S, Oklejewicz M (2003). The precision of circadian clocks: assessment and analysis in Syrian hamsters. Chronobiol Int.

[B5] Borjigin J, Li X, Snyder SH (1999). The pineal gland and melatonin: molecular and pharmacologic regulation. Annu Rev Pharmacol Toxicol.

[B6] Klein DC, Schaad NL, Namboordiri MA, Yu L, Weller JL (1992). Regulation of pineal serotonin N-acetyltransferase activity. Biochem Soc Trans.

[B7] Sun X, Deng J, Liu T, Borjigin J (2002). Circadian 5-HT production regulated by adrenergic signaling. Proc Natl Acad Sci U S A.

[B8] Arendt J (2006). Melatonin and human rhythms. Chronobiol Int.

[B9] Benloucif S, Guico MJ, Reid KJ, Wolfe LF, L'Hermite-Baleriaux M, Zee PC (2005). Stability of melatonin and temperature as circadian phase markers and their relation to sleep times in humans. J Biol Rhythms.

[B10] Honma K, Hashimoto S, Endo T, Honma S (1997). Light and plasma melatonin rhythm in humans. Biol Signals.

[B11] Klerman EB, Gershengorn HB, Duffy JF, Kronauer RE (2002). Comparisons of the variability of three markers of the human circadian pacemaker. J Biol Rhythms.

[B12] Lewy AJ, Cutler NL, Sack RL (1999). The endogenous melatonin profile as a marker for circadian phase position. J Biol Rhythms.

[B13] Duffy JF, Dijk DJ, Hall EF, Czeisler CA (1999). Relationship of endogenous circadian melatonin and temperature rhythms to self-reported preference for morning or evening activity in young and older people. J Investig Med.

[B14] Sun X, Liu T, Deng J, Borjigin J (2003). Long-term in vivo pineal microdialysis. J Pineal Res.

[B15] Borjigin J, Wang MM, Snyder SH (1995). Diurnal variation in mRNA encoding serotonin N-acetyltransferase in pineal gland. Nature.

[B16] Drijfhout WJ, Grol CJ, Westerink BH (1993). Microdialysis of melatonin in the rat pineal gland: methodology and pharmacological applications. J Neurochem.

[B17] Yamazaki S, Numano R, Abe M, Hida A, Takahashi R, Ueda M, Block GD, Sakaki Y, Menaker M, Tei H (2000). Resetting central and peripheral circadian oscillators in transgenic rats. Science.

[B18] Jones CR, Campbell SS, Zone SE, Cooper F, DeSano A, Murphy PJ, Jones B, Czajkowski L, Ptacek LJ (1999). Familial advanced sleep-phase syndrome: A short-period circadian rhythm variant in humans. Nat Med.

[B19] Barassin S, Saboureau M, Kalsbeek A, Bothorel B, Vivien-Roels B, Malan A, Buijs RM, Guardiola-Lemaitre B, Pevet P (1999). Interindividual differences in the pattern of melatonin secretion of the Wistar rat. J Pineal Res.

[B20] Gauer F, Poirel VJ, Garidon ML, Simonneaux V, Pevet P (1999). Molecular cloning of the arylalkylamine-N-acetyltransferase and daily variations of its mRNA expression in the Syrian hamster pineal gland. Brain Res Mol Brain Res.

[B21] Klein DC, Coon SL, Roseboom PH, Weller JL, Bernard M, Gastel JA, Zatz M, Iuvone PM, Rodriguez IR, Begay V, Falcon J, Cahill GM, Cassone VM, Baler R (1997). The melatonin rhythm-generating enzyme: molecular regulation of serotonin N-acetyltransferase in the pineal gland. Recent Prog Horm Res.

[B22] Wright KPJ, Groufier C, Duffy JF, Czeisler CA (2005). Intrinsic period and light intensity determine the phase relationship between melatonin and sleep in humans. J Biol Rhythms.

[B23] Czeisler CA, Duffy JF, Shanahan TL, Brown EN, Mitchell JF, Rimmer DW, Ronda JM, Silva EJ, Allan JS, Emens JS, Dijk DJ, Kronauer RE (1999). Stability, precision, and near-24-hour period of the human circadian pacemaker. Science.

[B24] Summer TL, Ferraro JS, McCormack CE (1984). Phase-response and Aschoff illuminance curves for locomotor activity rhythm of the rat. Am J Physiol.

[B25] Honma K, Honma S, Hiroshige T (1985). Response curve, free-running period, and activity time in circadian locomotor rhythm of rats. Jpn J Physiol.

[B26] Refinetti R (1999). Relationship between the daily rhythms of locomotor activity and body temperature in eight mammalian species. Am J Physiol.

[B27] Masubuchi S, Honma S, Abe H, Ishizaki K, Namihira M, Ikeda M, Honma K (2000). Clock genes outside the suprachiasmatic nucleus involved in manifestation of locomotor activity rhythm in rats. Eur J Neurosci.

